# *Cryptococcus* granuloma mimicking local progressed tumor after cryoablation of renal cell carcinoma: A case report^☆^

**DOI:** 10.1016/j.radcr.2022.06.084

**Published:** 2022-07-22

**Authors:** Hikari Fukui, Masashi Fujimori, Takashi Yamanaka, Naritaka Matsushita, Seiya Kishi, Chisami Nagata, Yuki Omori, Kouhei Nishikawa, Hiroto Yuasa, Hajime Sakuma

**Affiliations:** a Department of Radiology, Mie University School of Medicine, 2-174 Edobashi, Tsu, Mie 514-8507, Japan; b Department of Nephro-Urologic Surgery and Andrology, Mie University School of Medicine, 2-174 Edobashi, Tsu, Mie 514-8507, Japan; c Department of Pathology, Mie University School of Medicine, 2-174 Edobashi, Tsu, Mie 514-8507, Japan

**Keywords:** Cryoablation, Cryptococcus infection, Granuloma, Renal carcinoma, Pseudotumor, Renal granuloma

## Abstract

Infectious granulomas arising in the kidney are rare. However, there are few reports regarding renal granulomas, such as xanthogranulomatous pyelonephritis, sarcoidosis, malakoplakia, and tuberculosis. Here, we report a case of cryptococcal granuloma resembling a locally progressed tumor after percutaneous cryotherapy for renal cell carcinoma. A male patient in his 80s with rheumatoid arthritis underwent computed tomography (CT)-guided cryoablation for biopsy-proven papillary renal cell carcinoma. Follow-up contrast-enhanced CT imaging obtained 4 months after ablation confirmed an enhanced mass on the edge of the ablation zone. There were no symptoms related to the mass. This mass was radiologically diagnosed as local tumor progression and treated with repeated cryoablation. Percutaneous biopsy of the mass was performed immediately after the second cryoablation, and the mass was pathologically diagnosed as granuloma related to *Cryptococcus* infection. The patient was administered antifungal fluconazole for 1 year with a good outcome.

## Introduction

Renal cell carcinoma (RCC) ablation therapy is an alternative treatment for patients in whom surgical treatment is not indicated [1]. High-pressure gas-based cryotherapy has been widely available in the last decade, and reported local control rates were comparable with partial surgical resection or hyperthermal ablation of radiofrequency ablation [2].

**Fig. 1:**
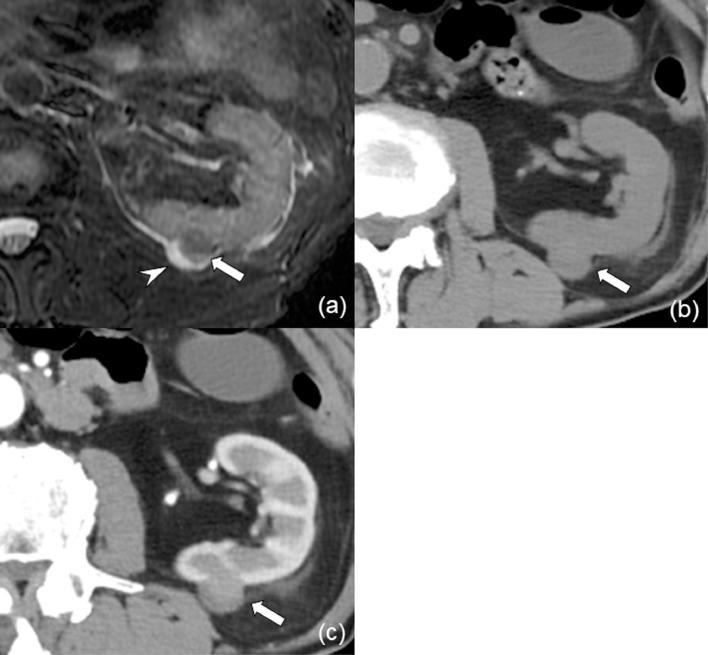
Images of the renal mass before initial cryoablation. (A) Fat suppression T2-weighted magnetic resonance image shows a homogeneous hypointense mass (arrow) on the dorsal border of the left kidney. The mass is adjacent to a renal cyst (arrowhead). (B, C) Computed tomography (CT) images of the mass. Contrast-enhanced CT image shows lower contrast enhancement of the mass (arrow) compared with the normal renal cortex in the corticomedullary phase.

**Fig. 2:**
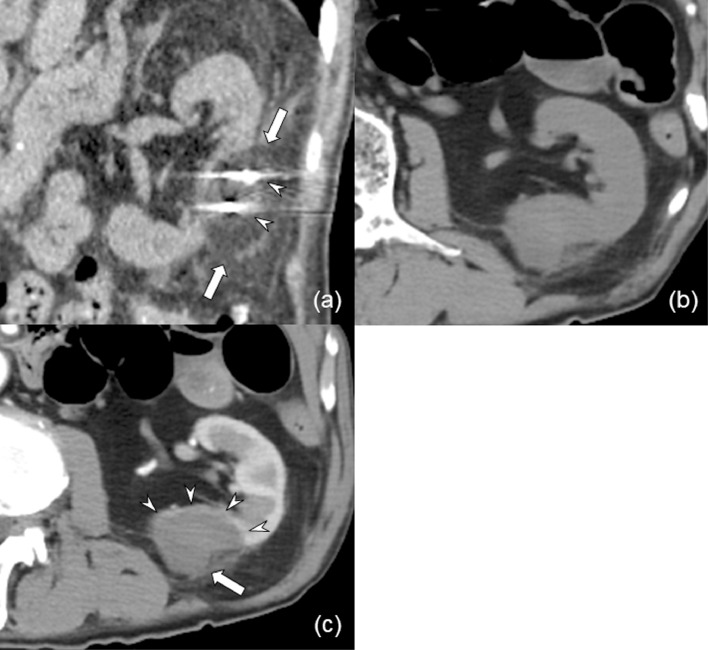
Sagittal reconstructed computed tomography (CT) image during initial cryoablation (CA). The renal mass on the left kidney is covered with low attenuation ice ball (arrows) with a sufficient margin. Arrowheads show 2 cryoprobes. (B, C) CT images obtained 2 weeks after initial CA. Contrast-enhanced CT image shows no enhancement of the mass (arrow) or ablation margin (arrowheads).

Imaging evaluation, such as computed tomography (CT) and magnetic resonance (MR) imaging plays an important role in both pre- and postablation follow-up. Approximately 20% of small renal masses could be nonrenal carcinoma lesions [3]. Generally, enhanced nodules that appear adjacent to the ablation zone are considered to be locally progressed tumors [4]; however, after cryotherapy, renal tumors could present with false-positive staining on enhanced image evaluation in 60% of cases [5]. Thus, imaging evaluation and interpretation should be performed carefully in this setting.

Granulomas arising in the kidney are rare. However, there are few reports regarding renal granuloma such as xanthogranulomatous pyelonephritis (XGP), sarcoidosis, malakoplakia, and tuberculosis [6,7].

In this report, we present our experience with cryptococcal granuloma resembling a locally progressed tumor after percutaneous cryotherapy for RCC.

## Case report

The institutional review board approved the preparation of this case report, and the patient provided informed consent for publication.

A male patient in his 80s with a history of rheumatoid arthritis had been taking prednisolone (Predonin,10 mg/day; Shionogi & Co., Ltd, Osaka, Japan) and methotrexate (Rheumatrex, 4 mg/day; Pfizer Japan Inc., Tokyo, Japan) for almost 14 years. During the follow-up, he was found to have a left renal mass with a maximum diameter of 1.6 cm in surveillance imaging for anemia. The renal mass was adjacent to a renal cyst on CT and MR imaging (Figs. 1A-C). On fat suppression T2-weighted MR image, the renal mass was homogeneous hypointense, and the mass showed lower contrast enhancement compared with normal renal cortex on corticomedullary phase contrast-enhanced CT (CECT) (Figs. 1A-C). The renal mass was radiologically diagnosed as RCC. Based on the tumor size and location, the patient was referred to our interventional radiology department for thermal ablation, where he underwent CT-guided cryoablation (CA) and was treated using two 17-gauge cryoprobes of IceRod (Galil Medical Ltd., Israel) with a CA system (CryoHit, Galil Medical Ltd., Israel). Two freeze-thaw cycles of 15 and 3 min were performed to cover the renal mass with an ice ball with a sufficient margin (Fig. 2A). For this renal mass, percutaneous biopsy was performed immediately after CA under CT fluoroscopy, and the mass was pathologically diagnosed as papillary RCC.

**Fig. 3:**
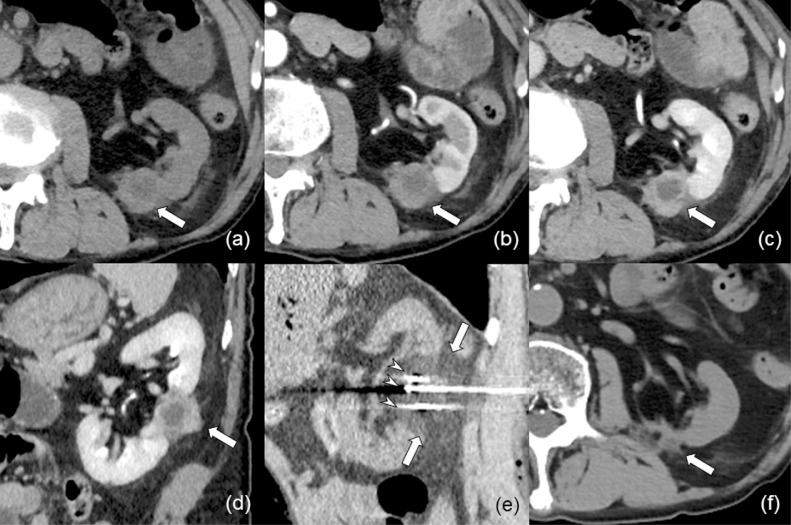
(A-D) Computed tomography (CT) images obtained 4 months after initial cryoablation (CA) confirmed a contrast-enhanced mass (arrow) on the edge of the ablation zone. The mass shows lower enhancement compared with normal renal cortex in both corticomedullary (B) and nephrographic (C) phase, and the mass has a poorly enhanced central part. (D) Sagittal reconstructed CT image on nephrographic phase. (E) Sagittal reconstructed CT image during the second CA shows that the renal mass is surrounded by low attenuation ice ball (arrows) with a sufficient margin. Arrowheads show 3 cryoprobes. (F) CT image obtained 11 months after the second CA shows remarkable shrinkage of the treated area (arrow).

Although CECT images obtained 2 weeks after CA showed no enhancement of the tumor (Figs. 2B and C), follow-up CECT imaging obtained 4 months after CA confirmed an enhanced mass of 2.2 cm in diameter on the edge of the ablation zone (Figs. 3A-D). MR imaging was not advisable because the patient had a metal clip on his colon for endoscopic polypectomy. There were no symptoms related to the mass. The mass was radiologically diagnosed as local tumor progression.

**Fig. 4:**
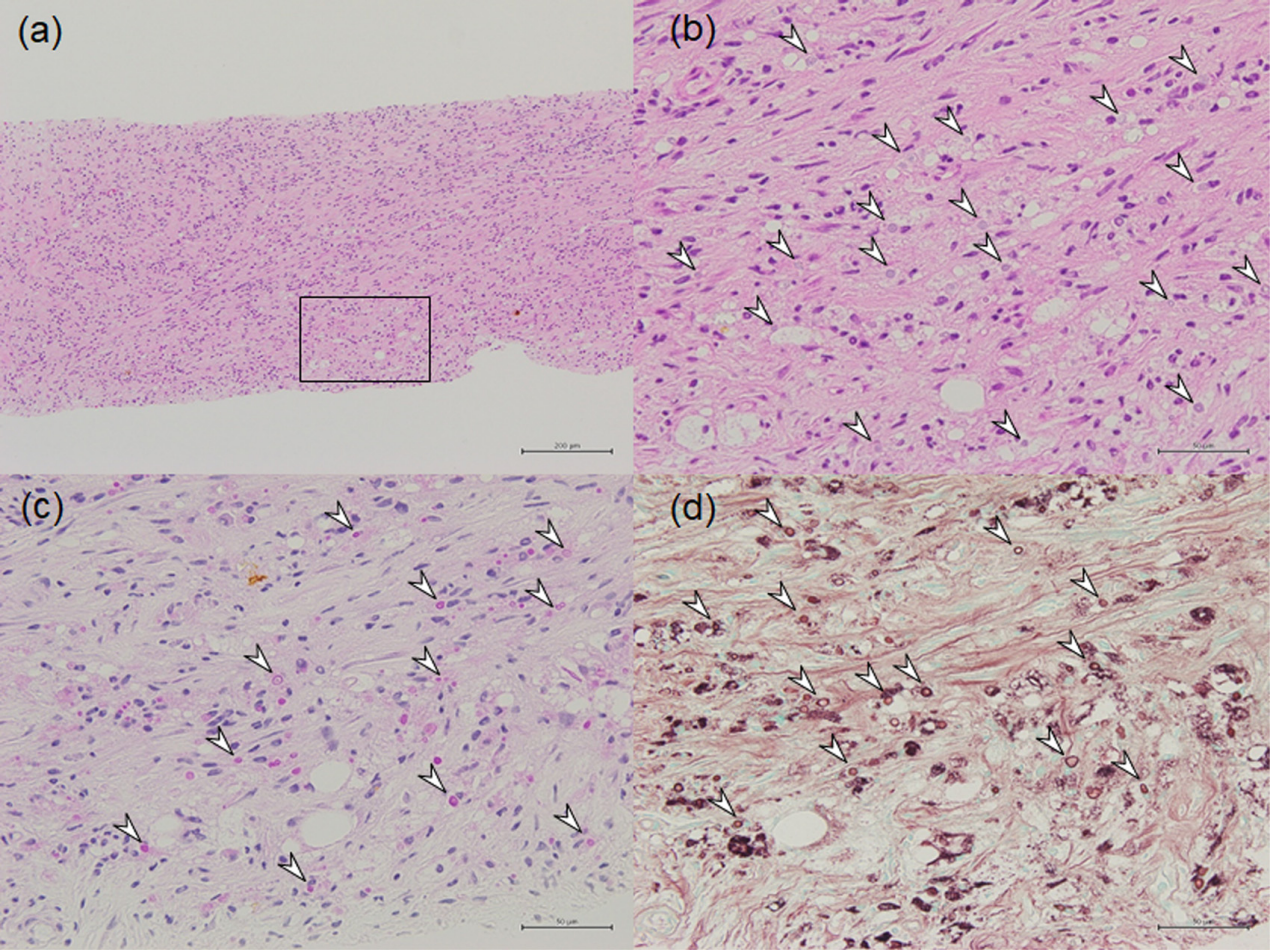
Histopathological images of a renal mass biopsy obtained immediately after the second renal cryoablation (CA). (A) Hematoxylin and eosin image in low magnification (×10). (B) High-magnification (×40) image of Box in (A) shows fibrous tissue with numerous yeasts (arrowheads are on some of them). Periodic acid Schiff (PAS) (C) and Grocott (D) staining in high magnification (×40). These round-shape yeasts were positively stained with PAS and Grocott staining (arrowheads are on some of them).

CA was repeated with 3 IceRod proves for treating this emerging contrast-enhanced mass in the same manner as the initial treatment 4 months after the initial CA (Fig. 3E). Considering the risk of tumor seeding, percutaneous biopsy of the mass was performed immediately after the second CA and revealed granulomas with fibrous tissue and numerous yeasts. The yeasts were positively stained with periodic acid Schiff (PAS) and Grocott staining and pathologically diagnosed as cryptococcosis. There was no neoplastic tissue in the specimen (Fig. 4). Blood test for glucuronoxylomannan (GXM) performed 2 weeks after the second CA was negative.

After a multidisciplinary discussion of infectious disease specialists, urologists, diagnostic radiologists, and interventional radiologists, this renal mass was diagnosed as a renal granuloma related to *Cryptococcus* infection and not a local progressed tumor. As this renal granuloma is considered a manifestation of disseminated cryptococcosis, a cerebrospinal fluid test, as well as brain MR imaging, was planned. However, cerebrospinal fluid test was declined, and no finding was suggestive of meningitis on MR images. Treatment using an antifungal agent against disseminated cryptococcosis was initiated. The patient was administered 400 mg of fluconazole (Fluconazole capsule, TAKATA Pharmaceutical Co., Saitama, Japan) daily. Four months later, fluconazole was reduced to 200 mg daily owing to decreased renal function and administered for 12 months. Enhanced CT images obtained 11 months after second CA showed remarkable shrinkage of the treated area (Fig. 3F) with no findings of RCC metastases or *Cryptococcus* dissemination.

## Discussion

In this case, histopathological examination of biopsies obtained from the CECT-enhanced foci that developed adjacent to the cryo-ablated zone of RCC was performed and the pathological diagnosis of *Cryptococcus* granuloma was reached.

After CECT, this mass was initially diagnosed as a locally progressed tumor. Due to the exophytic location of the mass, biopsies were obtained after the initial CA to reduce the possibility of tumor dissemination. The control rate of local progressed renal tumor with repeat ablative therapy is as high as 96.4%-99.0% [8]; thus, a second CA was planned in this case.

The reported diagnostic yield of the biopsied renal sample taken after CA is lower than that before ablation [9]; hence, renal nodule biopsy prior to the second CA might be beneficial for such cases. However, in this study, the biopsied renal specimen was not a nondiagnostic specimen (normal renal tissue, without renal tissue, or degenerated tissue), and there were no tumor cells.

To the best of our knowledge, renal *Cryptococcus* granuloma that developed after CA has not yet been reported. However, reported radiological findings of granulomatous renal masses such as XGP, sarcoidosis, and malakoplakia on CECT images that mimic RCC and biopsies were useful for confirming the diagnosis [6,10]. In our case, CECT images showed an enhanced nodule in the renal parenchyma with a poorly enhanced central area, and these findings also mimic other granulomatous masses shown above.

*Cryptococcus* infections usually develop among immunosuppressed individuals [11]. This patient has a long history of rheumatoid arthritis and has been taking prednisolone and methotrexate (2 immunosuppressive drugs) for 14 years, which is the most likely cause of his immunosuppressed condition and a risk factor for *Cryptococcus* infection and granuloma formation. The kidneys are usually not the primary sites of cryptococcal infection; thus, the strategy for this patient included systemic antifungal treatment against *Cryptococcus* infection. However, in this patient, no evidence of extrarenal *Cryptococcus* infection as cryptococcal meningitis or infection in other sites was found on CT or MR imaging. After consultation with an infectious disease specialist, the patient was treated with fluconazole for 1 year after the second CA. CT images obtained 11 months after the second CA showed remarkable shrinkage of the treated area. In the case of a pulmonary *Cryptococcus* infection, pulmonary nodules shrink with antifungal drugs [12]; however, in our case, it was uncertain whether the shrinkage of the treated area was caused by fluconazole or repeated CA.

## Conclusion

We present a case of renal granuloma caused by *Cryptococcus* infection mimicking local tumor progression after CA of RCC. Rebiopsy prior to percutaneous treatment could be beneficial even for renal masses that are radiologically diagnosed as local progressed renal cancer.
